# The Editor's Letter-Box

**Published:** 1917-06-16

**Authors:** Frederick Milner

**Affiliations:** Recuperative Hostels for Sailors and Soldiers Invalided from H.M. Services with Nerve-Strain (Registered under the War Charities Act, 1916), 20 Hanover Square, London, W.


Sir,—The writer of the article in your issue of the 19th ult. has failed to understand the object of our hostel, as much as he has, unintentionally no doubt, misrepresented my views as to the insane.

No disgrace, of course, attaches to the insane; on the contrary, I think they are to be pitied above all others. The disgrace I spoke of is attached to the Government, who could find no better refuge for its gallant men suffering from neurasthenia (the result of the terrible experiences they have gone through) than the lunatic asylum. A stigma, unfortunately, does attach to men who have been in an asylum, not because they have done anything disgraceful, but because they have been certified insane, and people are afraid that they may relapse, and so are chary of employing them. Therefore, if you send a man to an asylum who is merely suffering from neurasthenia, you do him an additional injury in this way.

The writer of the article asks, "Is it a fact that the unwounded but nerve-shattered man is cast off by the War Office, and has nothing done for him?" Yes, it is a fact, that can be very easily proved, that hundreds of our gallant men have been turned off uncured to become a burden to themselves and their families. Numbers have been invalided from the Services, certified, and sent to asylums, suffering not from madness, but from nerve-strain and exhaustion.

Our hostels are proving it now, for we have more applications than we can deal with at present. We have already had cases taken from asylums, and our doctors say these men show no signs of madness; all are suffering from neurasthenia, and all are in need of special medical care and individual treatment.

I have had most heartrending letters from these poor men saying that they are no more mad than I am, but that they will soon be driven mad if they continue to be herded with lunatics—I use their own expression. They ask if this is the only reward for all they have sacrificed for their country's sake.

It is true that after nearly three years of war the War Office seem to have realised to some extent the necessity for a reform, and with the help of Red Cross money they are starting a hostel at Golders Green, with 120 beds, for the reception of these unhappy men. And here is another proof of my assertion: they already have a long waiting list, some of whom, I hope, we shall be able to absorb into our hostels. If The Hospital wants any further proof, we shall have no difficulty in supplying it.

If The Hospital cares to send anybody down to our hostel at Hampstead, we shall warmly welcome them, and they can hear from the men themselves what they have suffered, and how hope has once again come into their lives since they have been with us. We do not object to a soldier being sent to an asylum if he is insane, though even then we say he should be sent as a private patient and be exceptionally treated—not sent as a pauper, as I am ashamed to say was the case in the early days of the war. We do object to a soldier being sent to an asylum when he is not insane, and we say that it is a lasting disgrace to the Government to send them there. We do not propose to take into our hostels men who are insane, but we do mean to take men who have been improperly sent to asylums, who are not insane.

These hostels have come to stay, and I am convinced they will accomplish a great and magnificent work. I hope no county in England will be satisfied to be without one.

I may mention a fact that will surprise The Hospital even more than the neglect to care for these sorely stricken men. Some of the men in our hostel, under this new Military Bill, were ordered to report themselves on June 4. I wrote to Mr. Macpherson in pretty vigorous language, but so far nothing has been done to stop this. You can imagine the effect such a notice has on men in this nervous condition; it is likely to undo much of our good work.—Yours very truly,

Frederick Milner.

Recuperative Hostels for Sailors and Soldiers Invalided from H.M. Services with Nerve-strain (Registered under the War Charities Act, 1916), 20 Hanover Square, London, W.

[We direct our correspondents' attention to the article on p. 207 dealing with these letters.—Ed. The Hospital.]

